# A new chicken 55K SNP genotyping array

**DOI:** 10.1186/s12864-019-5736-8

**Published:** 2019-05-22

**Authors:** Ranran Liu, Siyuan Xing, Jie Wang, Maiqing Zheng, Huanxian Cui, Richard P. M. A. Crooijmans, Qinghe Li, Guiping Zhao, Jie Wen

**Affiliations:** 1 Institute of Animal Sciences, Chinese Academy of Agricultural Sciences, No. 2 Yuanmingyuan West Road, Beijing, 100193 People’s Republic of China; 2 Animal Breeding and Genomics, Wageningen University &amp; Research, Wageningen, The Netherlands; 3 State Key Laboratory of Animal Nutrition, Ministry of Agriculture and Rural Affairs, Beijing, 100193 People’s Republic of China; 4 Key Laboratory of Animal (Poultry) Genetics Breeding and Reproduction, Ministry of Agriculture and Rural Affairs, Beijing, 100193 People’s Republic of China

**Keywords:** Chicken, Commercial line, Genotyping array, SNP

## Abstract

**Background:**

China has the richest local chicken breeding resources in the world and is the world’s second largest producer of meat-type chickens. Development of a moderate-density SNP array for genetic analysis of chickens and breeding of meat-type chickens taking utility of those resources is urgently needed for conventional farms, breeding industry, and research areas.

**Results:**

Eight representative local breeds or commercial broiler lines with 3 pools of 48 individuals within each breed/line were sequenced and supplied the major SNPs resource. There were 7.09 million - 9.41 million SNPs detected in each breed/line. After filtering using multiple criteria such as preferred incorporation of trait-related SNPs and uniformity of distribution across the genome, 52.18 K SNPs were selected in the final array. It consists of: (i) 19.22 K SNPs from the genomes of yellow-feathered, cyan-shank partridge and white-feathered chickens; (ii) 5.98 K SNPs related to economic traits from the Illumina 60 K SNP Bead Chip, which were found as significant associated SNPs with 15 traits in a Beijing-You crossed Cobb F2 resource population by genome-wide association study analysis; (iii) 7.63 K SNPs from 861 candidate genes of economic traits; (iv) the 0.94 K SNPs related to residual feed intake; and (v) 18.41 K from chicken SNPdb. The polymorphisms of 9 extra local breeds and 3 commercial lines were examined with this array, and 40 K - 47 K SNPs were polymorphic (with minor allele frequency &gt; 0.05) in those breeds. The MDS result showed that those breeds can be clearly distinguished by this newly developed genotyping array.

**Conclusions:**

We successfully developed a 55K genotyping array by using SNPs segregated from typical local breeds and commercial lines. Compared to the existing Affy 600 K and Illumina 60 K arrays, there were 21,41 K new SNPs included on our Affy 55K array. The results of the 55K genotyping data can therefore be imputed to high-density SNPs genotyping data. The array offers a wide range of potential applications such as genomic selection breeding, GWAS of interested traits, and investigation of diversity of different chicken breeds.

**Electronic supplementary material:**

The online version of this article (10.1186/s12864-019-5736-8) contains supplementary material, which is available to authorized users.

## Background

With a total of 107 chicken breeds, China has one of the richest local breed resources [1]. This diverse chicken genetic resource is a vital part of the diversity of biological genetic resources around the world and provides excellent material for breeding new varieties or to genetically improve breed.

China is the second-largest broiler producer and consumer all over the world, which accounts for approximately 11% of the chicken production across the globe (FAOSTAT, 2017). In China, chicken is the second largest meat product after pork, making up to 17% of the total meat production. Chicken meat is mainly obtained from the introduced white feather broilers and domestic yellow-feathered meat-type chickens (meat-type local chicken breed, meat-type bred variety and a relevant strain containing the consanguinity of Chinese indigenous chicken), each accounting for half of the consumption. However, the current challenge is how to effectively protect and maintain the existing local varieties. On the other hand, if breeding efficiency is promoted, new chicken lines breeding would be accelerated. The genome-wide SNP chip, also known as SNP array, arranges up to 25 million of DNA marker flanks on glass or special silicon chip to form the SNP probe array. It functions by means of the reaction of base pairing between the chip fixed DNA marker flanks with the target genome, so as to accurately identify the genetic information.

The genotyping arrays have been developed for pig [2], cow [3], dairy cattle [4], sheep [5], salmon [6], and buffalo [7] et al. In chicken, the first 3 K genotyping array was developed in 2005 with 3072 SNPs [8]. After that, in 2008, Groenen et al. did develop a 60 K bead chip for chicken which evenly covered the whole genome [9]. To date, the only available commercial arrays for chicken is Chicken the Affy 600 K SNP Array (Axiom Genome-Wide Chicken Genotyping Array), which was developed by Kranis et al [10]. The other arrays are privately owned by commercial companies. The array supplied an important tool for the genetic diversity analysis, breeds relationship analysis, GWAS, quantitative character positioning analysis of QTL, selective evolution investigation, and Genomic Selection [11]. Up till now the most efficient ways for SNP genotyping, biodiversity measuring, QTL mapping and genomic selection is using SNP arrays. These applications provide improved technical support for the conservation of indigenous breeds and development of new genetic lines/breeds.

One pitfall of all current chicken SNP arrays is the bias towards western commercial lines. The current chicken arrays, however, lack the genomic variation information of Chinese indigenous breeds. Therefore, it is imperative to develop a new type of genome-wide SNP chip with moderate flux in the chicken breeding industry, and also contains the genetic variation information specific to Chinese indigenous breeds. Overlap with the current arrays of the different platforms (Axiom and Illumina) is essential to link the commercial SNP arrays.

Through whole genome re-sequencing of a variety of Chinese native breeds and commercial chicken lines, integrating SNPs associated with economic traits detected in a crossing breed (either indigenous and commercial), a new public available moderate density (55 K) chicken array (IASCHICK) has been developed.

## Results

**Fig. 1:**
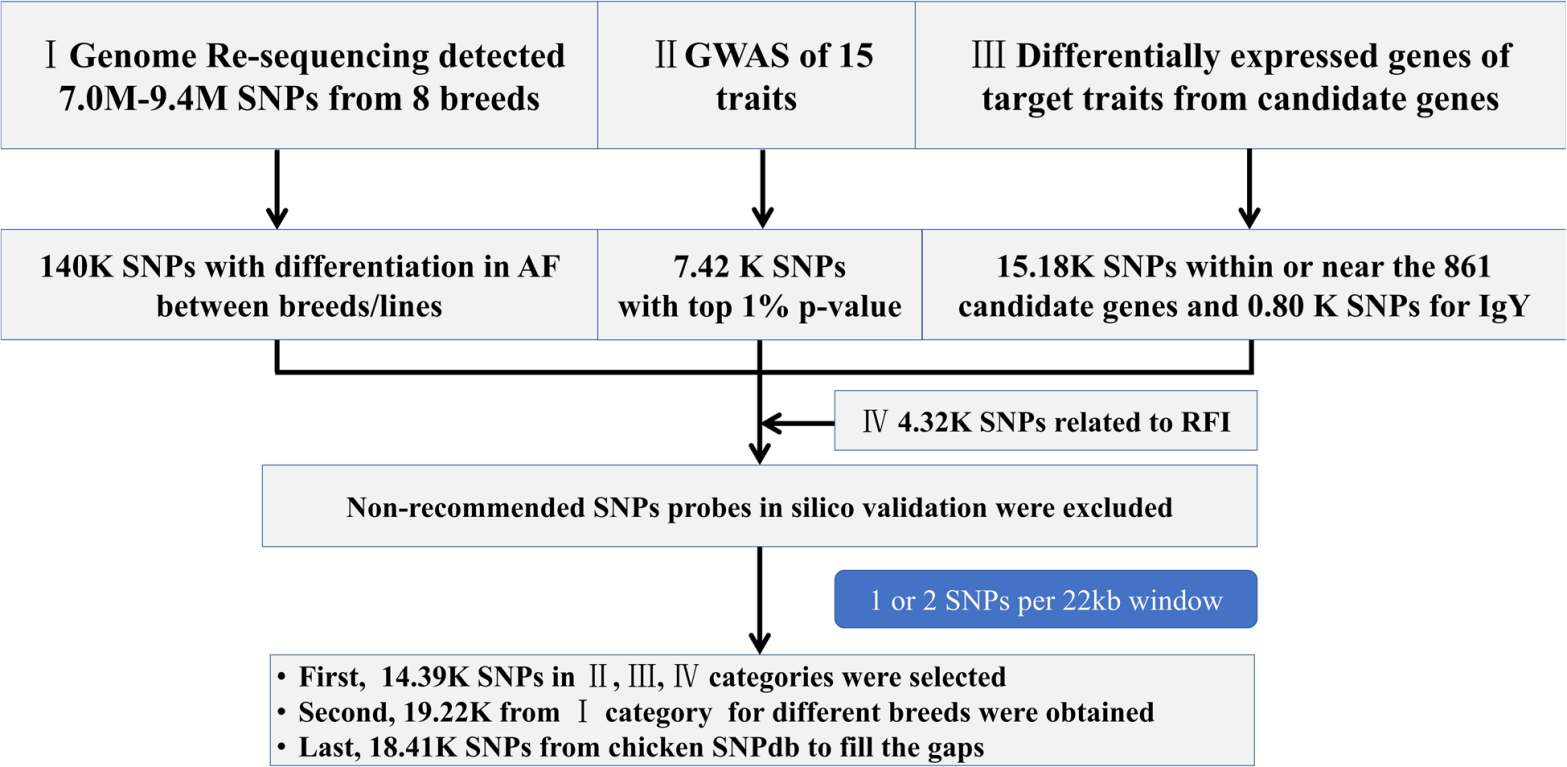
The roadmap for the design of the new chicken 55K SNP array

The SNPs selection was performed in four groups. The roadmap is shown in Fig. 1, and the establishment of the four groups are indicated in the following paragraphs.

### Genome re-sequencing of chickens supplying the first SNP group

Eight Chinese local chicken breeds or inbred lines were selected for whole genome sequencing. Each breed/line holds 3 pools of 16 individuals per library without individual barcodes (Table 1). The data summary of each library is provided in the Additional file 1. The number of SNPs per breed/line varied from 7.09 million to 9.41 million SNPs. The average number of detected SNPs was 8.61 M in the local lines, and 7.73 M in the commercial broilers. The total number of SNPs detected overall 8 breeds/lines was 15.2 M. The SNPs with minor allele frequency (MAF) &lt; 0.05 and with low ΔF were excluded for further steps. The 140 K SNPs, which allelic frequencies distinct to the control breeds, were subsequently used as the first group of candidate SNPs.

**Table 1:** Sequenced chickens and the number of SNPs detected from different breeds

Type	Breeds	Individuals in each pool^a^	No. of pools	Number of detected SNPs (with QC ≥ 20)^b^
Local Yellow-feathered chicken	Beijing-You	16	3	8,505,214
	Jingxing-Huang	16	3	8,349,627
	Sanhuang	16	3	9,405,319
Cyan shank partridge	Cyan shank partridge (fast growth rate)	16	3	8,954,795
	Cyan shank partridge (mediate growth rate)	16	3	8,884,232
Commercial white-feathered	Cobb maternal line	16	3	7,093,225
	Cobb paternal line	16	3	8,372,769
	Recessive White	16	3	7,556,464
	Total			15,312,402

^a^Each pool contained 8 males and 8 females

^b^Based on Gallus_gallus-4.0

### Selection of the second group of candidate SNPs based on the GWAS of 15 traits

The 7.42 K SNPs were demonstrated to have the top 1% genome-wide significance in 15 traits and were selected as the second group of SNPs. The details are shown in Additional file 2.

### Selection of the third group of candidate SNPs based on the genes associated with economic traits

SNPs in the regions of 861 candidate genes related to economic traits were used according to previous studies of gene/protein expression profiles. A total of 66.37 K SNPs in 383 genes for breast muscle and intramuscular fat development in embryonic and post-hatching periods [12], 24.69 K SNPs in 286 genes for body fat metabolism [13], 32.59 K SNPs in 146 genes for disease resistance [14], and 7.24 K SNPs in 46 genes that exhibited possible influence on other chicken economic traits [15] were selected (Additional file 3). The SNPs located in the 5 Kb of either side of the genes’ up- and down-stream were also considered.

According to the SNPs detected by the genome resequencing of the previously mentioned 8 breeds, 15.18 K candidate SNPs were selected from 118.47 K SNPs on all those genes, which had priority with mutations in exons, splicing regions, promotors, and the 3′ and 5′ untranslated regions (UTRs).

In addition, a batch of 798 SNPs from an unpublished capture sequencing of chicken chromosomes 11, 16, and 19 were included in the third candidate group (Additional file 4). The SNPs were significantly related to high IgY levels in Beijing-You and White Leghorn chickens.

There were 15.98 K candidate SNPs that were selected for the design of the final genotyping array.

### The fourth group of candidate SNPs are derived from whole genome sequences of low- and high-RFI chickens

Whole genome sequencing of low- and high-RFI chickens were performed to locate the genomic variants for RFI based on differences in allelic frequency between high- and low-RFI chickens as described in our previous study [16]. The selected 4.32 K SNPs (3.74 K RFI related SNP in Beijing-You chickens and 0.58 K RFI related SNPs in Cobb chickens) were used as the candidate SNPs for the design of the final genotyping array in the next step.

### Designing the Affy 55K genotyping array

Based on the above four groups of candidate SNPs, a custom-made algorithm was used to fix the final array. Finally, 52,184 SNPs were selected for the final array. The mean physical distance of SNPs in each involved chromosome shows in Table 2. The priority 1 SNPs (the SNPs in group 2, 3 and 4) and 25 INDELs were first placed on the final SNP panel. The next step was addition of the priority 2 SNPs (the SNPs in group 1). The remaining 18.41 K SNPs was selected for the blank windows in the whole chicken genome which the SNPs in the four groups cannot be covered.

**Table 2:** The number of SNPs of the 55K array on each chromosome and their distance^a^

Chromosome	Number of SNPs	Mean Distance (K bp)
1	10,228	19.21
2	7077	21.14
3	5196	21.44
4	4589	19.91
5	2705	22.10
6	1750	20.36
7	1684	21.63
8	1314	22.81
9	1236	18.98
10	1399	14.59
11	1373	14.67
12	1389	14.34
13	1041	17.67
14	1118	14.43
15	761	16.71
16	81	7.37
17	724	14.31
18	736	14.94
19	725	13.74
20	867	16.04
21	503	13.53
22	153	30.00
23	321	17.76
24	390	15.98
25	106	26.67
26	339	15.36
27	277	20.22
28	317	15.91
Z	3785	21.67
Summary	52,184	

^a^ The distance between SNPs based on Gallus_gallus-5.0

**Fig. 2:**
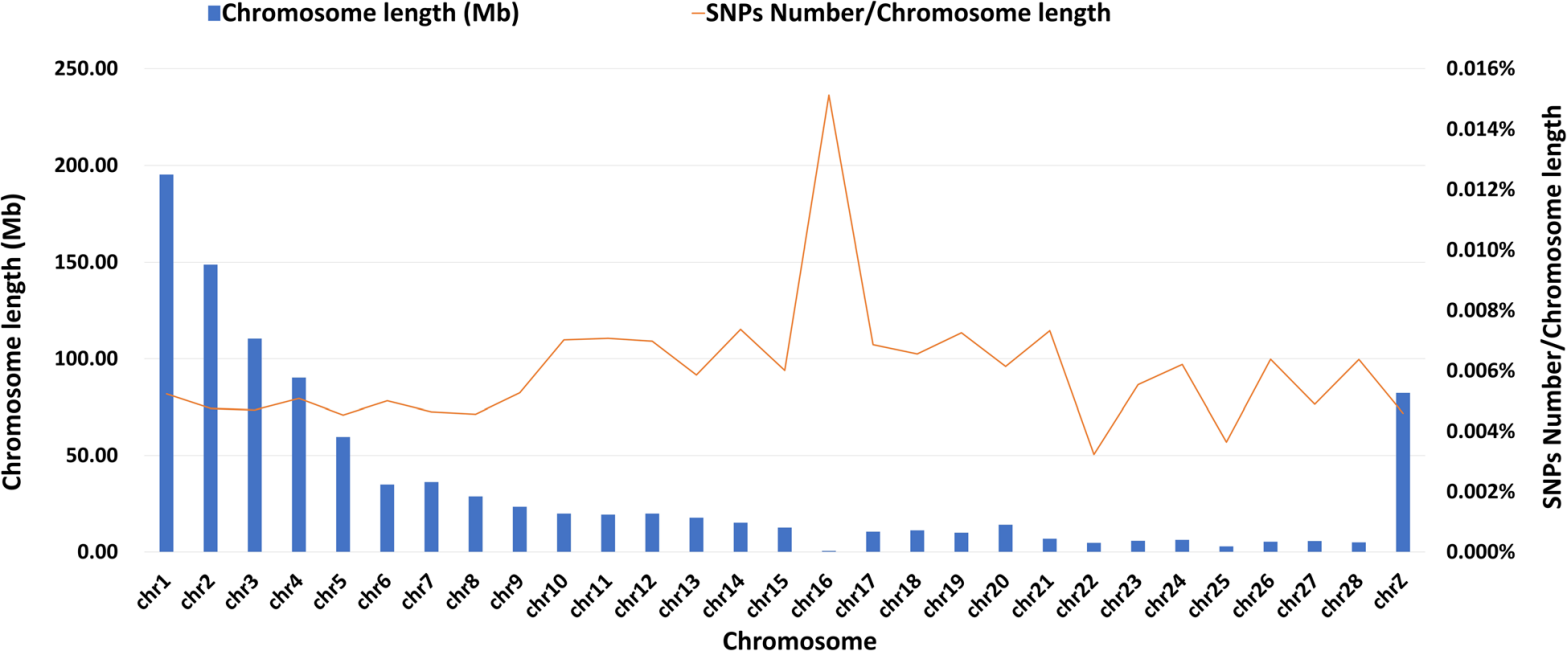
The chromosome-wise SNP density of the 55K SNP array. Chromosome length shows in left axis (based on galGal-5) and SNP density shows in right axis

The SNPs positions of 55K array were given in Additional file 5. The selected SNPs were derived from the following five groups (Table 3): (i) 19.2 K SNPs from whole genome sequencing of the eight chicken breeds/lines; (ii) 7.42 K trait-related SNPs from the Illumina 60 K SNP Bead Chip, which were found as SNPs significantly associated with 15 economic traits; (iii) 15.98 K SNPs from 861 candidate genes of target traits and high IgY level related region; (iv) 4.32 K SNPs related to chicken RFI; and (v) 18.41 K from chicken SNPdb. In the final genotyping array, 99.85% of SNPs could be annotated (Table 4). The distribution of SNPs on the chromosomes is shown in Fig. 2.

**Table 3:** The number of SNPs from five candidate groups in the final 55K array

Resource Category	Number of SNPs in 55K array
I. Genome Re-sequencing of eight breeds	
White-feathered	12,555
Yellow-feathered	3940
Cyan-shank Partridge Chicken	2724
II. SNPs based on GWAS of 15 traits	5980
III. SNPs on the candidate genes	7630
IV. SNPs related to RFI	943
V. SNPs from chicken SNPdb	18,412
Total	52,184

**Table 4:** Summary of the SNPs effect prediction in 55K array

Item	Count	Percent (%)
Total number of SNPs in the panel	52,184	
Annotation possible	52,108	99.85
intergenic variant	16,106	30.86
intron variant	25,275	48.43
intron variant &amp; noncoding transcript variant	3981	7.63
missense variant	590	1.13
missense variant &amp; splice region variant	13	0.02
synonymous variant	1601	3.07
Splicing	187	0.36
start/stop gained/lost/retained	12	0.02
3 prime UTR variant	1358	2.60
5 prime UTR variant	229	0.44
upstream gene variant (1 kb)	871	1.67
downstream gene variant (1 kb)	1014	1.94
noncoding transcript exon variant	871	1.67

### The comparisons of the Affy 55K array with the existing chicken arrays (Affy 600 K array, and Illumina 60 K)

**Fig. 3:**
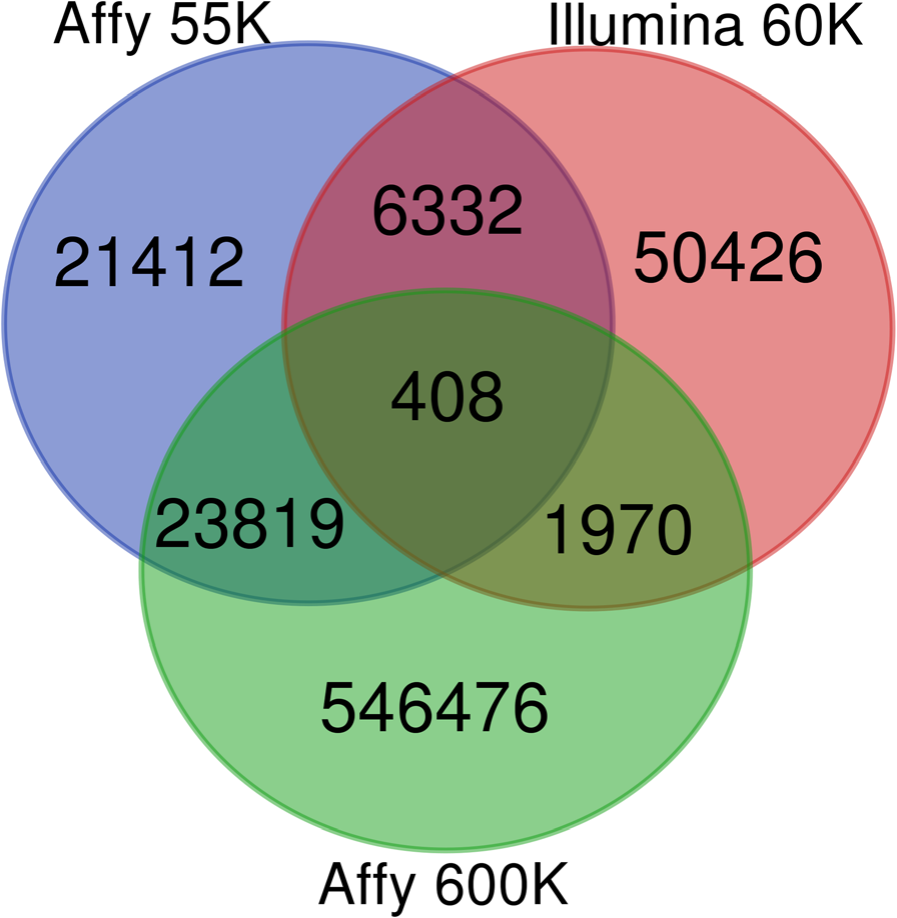
The comparison of the overlap of the SNP positions among Affy 55K array, Affy 600 K array and Illumina 60 K array

All the SNPs of this 55K array, Affy 600 K array [10], and Illumina 60 K array [9] were mapped to the latest chicken genome (GRCg6a). The overlap of the 3 arrays is shown in Fig. 3. There are 6740 SNPs (13%) which overlap between the Affy 55K array and the Illumina 60 K array. When comparing to the Affy 600 K array, there are 24,227 SNPs that overlap between the 55K array which accounts for 46%. There were 21,412 new SNPs included in 55K array compared to the existing arrays.

### Validation of the 55K array in 13 chicken breeds/lines

All samples from 10 Chinese local breeds (Chahua, Dagu, Liyang, Luhua, Qingyuan, Silkie, Wenchang, Bai’er, Xianju, and Jingxing-Huang) and 3 commercial lines (Hubbard, Cobb, and White Leghorn) were genotyped.

The average call rate for each breed ranged from 97.0% (Qingyuan) to 98.7% (Cobb). Across all populations, 76.7 to 88.0% of the 52.18 K SNPs were polymorphic, with MAF ≥ 0.05. The average MAF ranged from 0.22 (Bai’er chicken) to 0.27 (Wenchang chicken) (Table 5).

**Table 5:** Number of polymorphic loci in local breeds and introduced lines

Breeds	Average Call Rate (%)	Polymorphic loci^a^	Mean MAF^b^
Chahua	97.56	42.3 K	0.242
Qingyuan	97.00	45.2 K	0.267
Wenchang	97.10	46.5 K	0.277
Luhua	97.21	44.4 K	0.261
Liyang	97.23	40.4 K	0.229
Dagu	97.46	43.7 K	0.253
Bai’er	97.36	40.0 K	0.222
Xianju	97.13	40.1 K	0.235
Silkie	97.03	45.0 K	0.258
Hubbard	97.88	46.1 K	0.269
Cobb	98.70	45.2 K	0.237
White Leghorn	97.99	43.5 K	0.249

^a^MAF &gt; 0.05,

^b^Across all 52.2 K loci

**Fig. 4:**
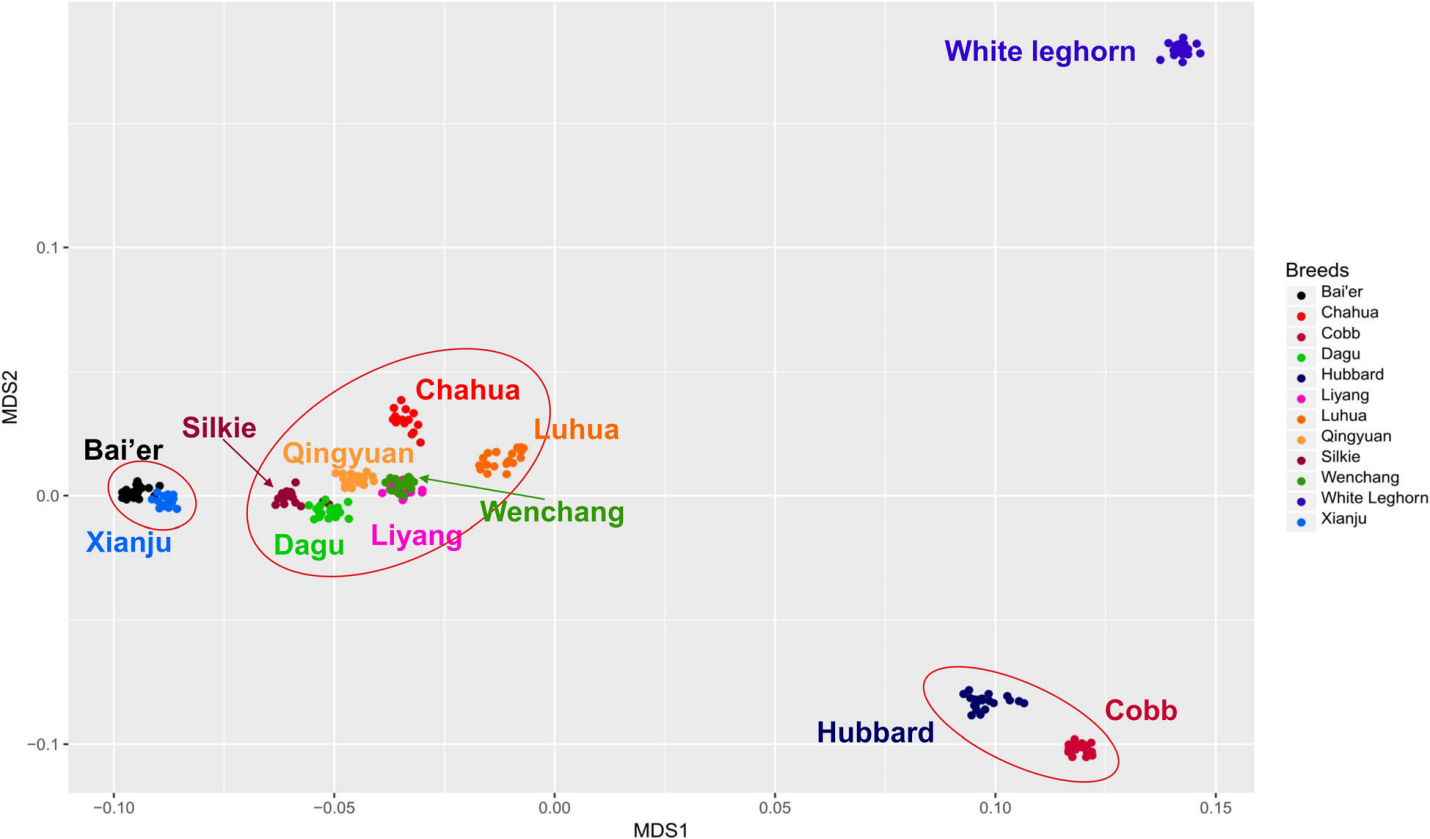
Results of multidimensional scaling analysis of 12 breeds/lines. The scatters show the individuals’ position in the MDS plot, different colors represent different breeds/lines

An MDS analysis was performed using the genotyped data to investigate the ability of the 55K panel to detect population stratification in the validated samples. Figure 4 shows the relative coordinates of individuals when plotted using the two largest principal components. Individuals originating from the commercial broilers, Hubbard and Cobb tightly clustered. The Chinese indigenous meat-type breeds clustered together. The two Chinese indigenous egg-type breeds, Xianju and Bai’er, clustered together. The remaining local breeds (mainly characteristic of meat-types) were located relatively close to each other compared to egg-type breeds and commercial broilers. The commercial layer White Leghorn chickens were placed relative far away from to the Chinese local breeds and commercial broilers in Fig. 4.

**Fig. 5:**
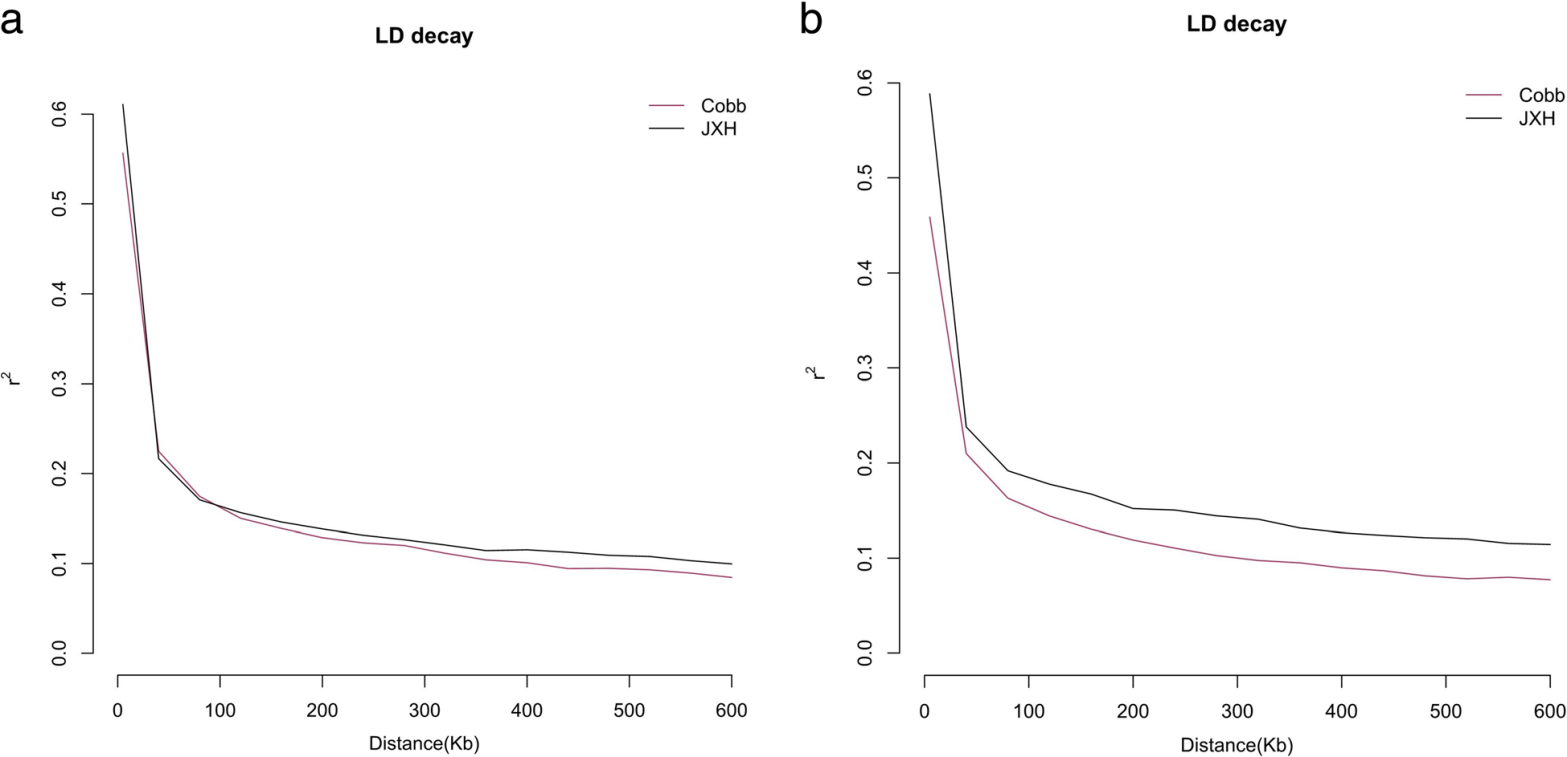
The LD decay plots. (**a**). from the Cobb and Jingxing-Huang (JXH) chickens in chromosome 1; (**b**). from Cobb and JXH chickens in chromosome 2

The linkage disequilibrium (LD) in Jingxing-Huang chicken and Cobb paternal line chicken were calculated, respectively. Figure 5 a and b shows the LD decay of the Jingxing-Huang and Cobb paternal lines for chromosome 1 and 2, respectively. The average levels of LD between adjacent SNPs in Jingxing-Huang breeds of chromosome 1 is 0.61 and for chromosome 2 is 0.58, whereas in the Cobb line these LD levels are 0.56 and 0.46, respectively. The mean LD level decay is around 0.22 in 40 Kb. The r^2^ of LD in the Jingxing-Huang chickens are larger than that in Cobb paternal line. Additional file 6 and Additional file 7 showed the r^2^ of LD decreasing with the increased SNPs distance in the two populations in whole genome level.

## Discussion

The 52.18 K SNPs selection was performed in four groups and NCBI SNPdb using several criteria. The first group includes 140 K SNPs screened in genome sequences of eight Chinese local and commercial chickens. The Beijing-You chicken and Guangxi Sanhuang chicken are representative of Chinese yellow-feathered chickens, which possess excellent meat quality and flavor [17]. Two Cyan-shank partridge lines possess meat flavor and appearance that are usually chosen by consumers. The Jingxing-Huang line is widely used in local breeding programs because of its dwarfism, feed-saving and space-saving characteristics [18]. The Cobb paternal line is a type of fast-growing line. The Recessive White chicken is a fast-growing line, which is popular in Chinese breeding programs because it can improve the growth rate of commercial generations without changing the appearance of offspring, when crossed with local colorful breeds. The SNPs with high ΔF between the local breeds and the commercial lines were used to determine the polymorphisms that have a larger difference in allelic frequency between breeds/lines. The main aim of whole-genome sequencing of different chicken breeds is to detect SNPs, although the pooled sequencing might generate potential bias.

The second, third and fourth groups are those potentially associated with economic traits, including 7.42 K SNPs associated with weight, carcass, immune and meat quality traits, 15.98 K SNPs for breast muscle, body fat, reproduction *etc*. Improving feed efficiency is an important goal in poultry industry to reduce costs. RFI was considered independent of body weight and weight gain, selection for RFI would improve the feed efficiency without changing the economic traits [19]. For special interests, 4.32 K SNPs were selected from a whole genomic sequencing research of low- and high- RFI Cobb and Beijing-You chickens. The strategy for the first selection of SNPs in candidate genes for the array is that these SNPs have a higher potential to be in LD to the causative SNPs for the target traits. Finally, the last 18.41 K SNPs were selected from chicken SNPs database to make all SNPs cover the whole genome evenly. The average distance among SNPs are 22 kb generally (Fig. 2). Due to specific selection on immune related genes, a high SNP density in chromosome 16 is observed. Based on the limited known information and substandard assembly (galGal-5.0) of the micro-chromosomes, we would not obtain SNPs of insufficient quality in the micro-chromosomes. In summary, the setting of the algorithm was to select SNPs with relevant function and even distribution across the genome in terms of physical distance and obtained the representation of SNPs in local or commercial breeds.

When comparing to the Affy 600 K array, there are 24,227 SNPs that do overlap with the 55K array which accounts for 46%. The reason for this high percentage of overlap is that the 18.41 K SNPs for filling the gaps in the whole chicken genome were selected from chicken SNPdb, and the probe validated SNPs hold a high priority. This result showed that there were 21,41 K new SNPs included on the Affy 55K array compared to the two existing arrays. The results indicate that imputation of the 55K genotyping data to the high-density SNPs genotyping data is possible. In the new 55K genotyping array, 69% of SNPs are within genes (non-intergenic variant), the proportion is higher than the proportion in the Affy 600 K array (54%), and lower than the proportion in Illumina 60 K array (86%).

To investigate the ability of our 55K panel to detect polymorphisms and population structure in local or commercial breeds/lines. Nine Chinese local breeds (Chahua, Dagu, Liyang, Luhua, Qingyuan, Silkie, Wenchang, Bai’er, and Xianju) and 3 commercial lines (Hubbard, Cobb, and White Leghorn) were tested. The average call rate for each breed ranged from 97.0% to 98.7%. Across all populations, 76.7% to 88.0% SNPs were polymorphic (Table 5), which indicates that the 55K genotyping array can be used to determine genetic variation both in various local Chinese breeds and in commercial meat-type and egg-type breeds.

According to the results of MDS analysis (Fig. 4), individuals originating from the commercial broilers, Hubbard and Cobb clustered together tightly and the two Chinese indigenous egg-type breeds, Xianju and Bai’er, clustered together. It might be due to the fact that the two breeds were selected in the same direction [1]. This result was supported by the previous study on the genetic diversity of Chinese domestic fowls by mtDNA analyzing: the inter-population net nucleotide divergence (Da) between Xianju and Bai’er was 1.006, which was lower than the Da (1.115) between Xianju and Dagu chickens [20]. The remaining local breeds (mainly characteristic of meat-types) were located relatively close to each other compared to egg-type breeds and commercial broilers. The commercial layer White Leghorn chickens were placed relative far away from to the Chinese local breeds and commercial broilers. The relative proximity of Chinese local meat-type chicken and Chinese egg-type chicken in the MDS plot might be due to their shared region and ancestry. Thus, the MDS results were in agreement with the existing knowledge of the lines/breeds and were also in similar to the previous studies, which showed phylogenetic relationships among different chicken breeds [1].

The linkage disequilibrium (LD) in the Jingxing-Huang breeds and Cobb paternal line were calculated and compared. The mean LD level decay to around 0.22 in 40 Kb. This result is similar with the result of Fu et al. in 2015 [21]. The r^2^ of LD in the Jingxing-Huang breed is larger than that in Cobb paternal line. The Jingxing-Huang is an inbred line has a relatively small effective population size whereas the Cobb paternal line is three to four times larger.

In China, indigenous yellow-feathered chickens are highly diverse (more than 100 local breeds and 70 crosses). The major obstacle in applying genomic selection for improvement of local breeds is the cost of genotyping array. The 55K array has a medium SNPs density, cost-efficient, and optimal for Chinese local breeds compared with the existing 600 K commercial array. Furthermore, the 55K genotyping array incorporated known SNPs loci that possess a high potential for association with economic traits and traits that are expensive and difficult to measure, which will be interesting for both GWAS and genomic selection (GS) projects.

With the rapid development of next-generation sequencing technologies and reduction of the costs, genotyping with re-sequencing (IBS) will be the focus of future research. In the current phase, however, the IBS system is more complex and not as solid as the SNP array. The array genotyped data can be easily analyzed and standardized according to constant array SNP positions. The batch effect can be excluded by different laboratories and companies.

## Conclusions

In conclusion, we developed Affy 55K genotyping array that was designed to use SNPs that are segregated in Chines local chicken breeds and commercial lines/breeds, and where large number of SNPs are associated with economic traits. Compared to the existing Affy 600 K and Illumina 60 K arrays, 21,41 K new SNPs were included in the 55K SNP array. The results from the our Affy 55K genotyping array can be imputed to the high-density SNPs genotyping data. This array offers wide range of potential applications, such as the evaluation of germplasm resources of chicken breeds, investigation of diversity of different chicken breeds, implementation of genome-wide association studies and genomic selection.

## Methods

### Animals

For whole genome sequencing, the 384 chickens were sampled from eight local breeds or inbred lines (Table 1). Chickens were supplied by Institute of Animal Sciences in CAAS (local breed Beijing-You, inbred Jingxing-Huang line), Jiangsu Lihua Co. Ltd. (Cyan-shank Partridge lines with fast and mediate growth rates, respectively), Institute of Poultry Sciences of CAAS (Sanhuang chicken and Recessive White chicken), Xinguang Nongmu Co. Ltd. (paternal and maternal line of Cobb in parental generation). In addition, a set of 15 to 21 chickens in each breed/line were used for SNP array evaluation, which were sampled from 9 local breeds and 3 commercial lines. Chickens were supplied by the Institute of Poultry Sciences of CAAS (Bai’er chicken, Chahua chicken, Dagu chicken, Liyang chicken, Qingyuan chicken, Silkie, Wenchang chicken, Luhua chicken and Xianju chicken), Xinguang Nongmu Co. Ltd. (paternal lines in parent generation from Cobb and Hubbard), the Institute of Animal Sciences of CAAS (White Leghorn). Two groups with 87 and 100 chickens from Jingxing-Huang and Cobb were also used for SNP array evaluation. The blood samples used in this study were all collected from chickens under the veterinary supervision and the Guidelines for Experimental Animals established by the Ministry of Science and Technology (Beijing, China), and with the approval of Animal Ethics Committee of the Institute of Animal Sciences. No anaesthesia or euthanasia methods were used. There was no evidence at health examination that any of the involved chickens had clinical diseases caused by the sampling.

### Whole genome re-sequencing

Genomic DNA was isolated from blood samples by the phenol-chloroform method. Samples DNA quality were validated by gel electrophoresis and Nanophotometer. The individual DNA samples (48 from each breed/line) were pooled to construct three libraries, with each library containing 8 males and 8 females. The libraries were constructed using the Nextera DNA Library Preparation Kit (Illumina Inc., San Diego, CA) according to the manufacturer’s standard protocol. All libraries were sequenced on the Illumina Hiseq2500 (2 × 125 bp).

### Genome sequence alignment and detection of the first group of candidate SNPs

Reads were filtered for low quality (&gt; 10 consecutive nucleotides with Phred scores &lt; 10), adaptor sequences, and sequences without a quality control-passed paired read using NGSQC toolkit (v2.3.3) [22]. Each trimmed pool sequencing coverage are shown in Table S5. Filtered sequenced reads were mapped to the reference genome (Gallus_gallus_4.0) by BWA software (v0.7.10) [23]. PCR duplications were removed with -rmdup argument in Samtools (version 0.1.1.18) [24]. SNPs were identified and genotyped for each data set with mpileup function in Samtools, then called by VarScan [25]. Only those highly confident variants supported by both methods were kept for downstream analyses. The SNPs calling details parameter were described by Liu et al [16]. The SNPs with MAF &lt; 0.05 and the INDELs in each breed/line were filtered by vcftools [26]. In Beijing-you chicken, Jingxing-Huang chicken, Sanhuang chicken, and the two lines of cyan-shank partridges minus the MAFs of Cobb paternal line, as well as the MAFs of Recessive White chicken, and the paternal and maternal generation of Cobb minus the MAFs of Beijing-You chicken, respectively. The SNPs with low ΔF were excluded. The value of ΔF was adjusted for 140 K SNPs reserved in local breeds and commercial lines to generate the first group of candidate SNPs. The threshold of △F in local breeds and commercial lines are 0.609 and 0.731, respectively. The SNPs acquired through genome re-sequencing of eight breeds/lines supplied the major data for the first group of SNPs in the array. SNPs specific for chromosome W were removed and were not considered in current designing. There are also 25 INDELs for special interest, which were defined as priory 1.

### Selection of the second group of candidate SNPs based on GWAS analysis of 15 traits

The second group of candidate SNPs was selected according to a GWAS analysis of 15 traits. Phenotype and genotype data were generated from the CAAS chicken F2 resource population as described in Sun’s report [27]. Briefly, the population was derived from a cross between local Beijing-You chickens and commercial Cobb broilers (Cobb-Vantress, Inc.). The weight, carcass, immune and meat quality traits were measured from 367 F2 chickens. The 15 traits were as follows, (a.) body weight of day 28 and day 42, (b.) carcass traits including total weight percentage after slaughtering, breast muscle weight percentage, leg muscle weight percentage, abdominal fat percentage, (c.) meat quality traits including the breast muscle intramuscular fat ratio, ultimate pH (24 h), meat lightness, redness value and yellowness value of breast muscle, (d.) immune traits including IgY level to sheep red blood cell, the heterophil and lymphocyte ratio, IgY level in serum, and the average red blood cell backlog.

SNPs were genotyped by using Illumina 60 K SNP Bead chip for chicken [9]. All description of the phenotypes had been reported by Sun et al. in 2013 [27]. To maximize the polymorphism resources for SNP array, the GLM procedures were used for the GWAS analysis and was performed by PLINK software (version 1.07) [28] with 42,585 SNPs passed quality control. The details were described by Sun et al. [27]. The SNPs with top 1% lowest *p*-values were used in the following procedures.

### Selection of the third group of candidate SNP based on the associated genes for target traits

Known candidate genes for economic traits were collected and used for the SNP array design. All genes were identified through previous researches by our group [12, 13, 29, 30]. We retrieved total 861 genes related to skeletal muscle and intramuscular fat development, chicken fat metabolism, salmonella enteritidis resistance etc. (Additional file 2). The SNPs were annotated by the Ensembl tool VEP [31]. Mutations and the SNPs in the exons, splicing region, and UTRs were firstly selected out. A maximum of 5 candidate SNPs were selected out for each gene.

In addition, the SNPs in this group also included a batch SNPs detected from a set of capture sequencing of Chr. 11, Chr. 16, and Chr. 19 of White Leghorns and Beijing-You chickens with low or high serum IgY (Liu et al., unpublished, Supplement Table S3).

### Selection of the fourth group of candidate SNPs for RFI

The fourth group candidate SNPs were selected from a whole genomic re-sequencing research of low- and high- RFI Cobb and Beijing-You chickens. SNPs calling results showed that 8,505,214 and 8,479,041 single nucleotide polymorphisms (SNPs) were detected in low- and high-RFI Beijing-You chickens, respectively; 8,352,008 and 8,372,769 SNPs were detected in low- and high-RFI Cobb chickens, respectively. The SNPs with Fst value &lt; 5% in each breed were excluded followed by SNPs with mean ΔF &lt; 0.35 between low- and high-RFI chickens. Through the above filtering processes, 3.74 K SNPs assigned to 1137 candidate genes in Beijing-You chickens and 0.58 K SNPs (448 genes) in Cobb chickens were reserved [16].

### Selection of the SNPs from chicken SNPs database

The first four groups cannot cover the whole genome evenly. In the fifth group, SNPs were selected from chicken SNPs database from NCBI (ftp://ftp.ncbi.nih.gov/snp/organisms/archive/chicken_9031/).

### SNP screening according to the scoring of probes

All the SNPs’ positions were transformed from WASHUC2.1 (Illumina 60 K), and Gallus_gallus-4.0 (Affy 600 K) to Gallus_gallus-5.0 (Affy 55 K) by the LiftOver tool on UCSC Genome Browser. Take utility of all SNPs from the five candidate groups above, in silico validation, was performed using the AxiomGTv1 algorithm of APT, which generated an output score file containing p-convert values, signifying the SNP array quality and list of recommended and non-recommended SNP probes. For a high-quality SNP array design, non-recommended SNP probes were all excluded in the following procedure.

### SNPs selection procedure for the final 55K array

The final SNPs selection was done in multiple steps using several criteria. The roadmap is shown in Fig. 1.

A custom-made algorithm was applied as described below. According to the Gallus_gallus-5.0, the chicken genome length is about 1.2 Gb. To ensure the probe position evenly distributed in the chicken genome, the whole genome was distributed by windows with 22 Kb length. The backward window started from the probe position of the forward probe position. The selection of the final array was performed on each chromosome separately. The first four groups SNPs were divided as 2 priorities. The SNPs in group 2, group 3, group 4, and the INDELs in group 1 were defined as priority 1, and the SNPs in group 1 were defined as priority 2.

a) The selection of the SNPs in priority 1. If there is no SNP in a 22 kb window, the window will be reserved. b) If there are one or two SNPs in the window, the SNP(s) was reserved. c) If there are 3 or more SNPs in a window, only 2 SNPs in this window will be reserved, which can make the SNPs even distributed in this window according to the following formula. SD^2^= $\frac{{\left(\mathrm{S}-\overline{\mathrm{x}}\right)}^2+{\left({N}_i-\overline{\mathrm{x}}\right)}^2+{\left({\mathrm{N}}_{\mathrm{j}}-\overline{\mathrm{x}}\right)}^2+{\left(E-\overline{\mathrm{x}}\right)}^2}{4}$. In the formula above, the S and E are the start position and the end position of the window respectively; and N_i_ and N_j_ are the target SNPs position in the window. The SNPs N_i_ and N_j_ which can minimum the SD^2^, will be reserved.
The selection of priority 2 SNPs. The windows reserved 1 or 2 SNPs will be skipped. The windows without SNP will be filled by one SNP of priority 2 according to the formula described above.
The windows without any SNP will be filled by 1 SNP from the NCBI SNPdb of chicken, while the validated SNPs will have a priority for filling.

The final array contains 55K probes for 52 K SNPs, which were manufactured by Affymetrix® using photolithography. The redundant probes are used for interrogating each SNPs [32, 33]. The final 52 K SNPs were annotated by the online tool Ensembl VEP [34].

### The comparisons of the 55K Affy array with the existing arrays (Affy 600 K array, and Illumina 60 K)

All the SNPs’ positions were transformed from WASHUC2.1 (Illumina 60 K), Gallus_gallus-4.0 (Affy 600 K) and Gallus_gallus-5.0 (Affy 55 K) to GRCg6a by the LiftOver tool on UCSC Genome Browser. All the SNP positions of the three genotyping arrays were compared. The SNPs on 600 K array and 60 K array were also performed by Ensembl VEP [31]. Overlapping Venn plot was performed by the Calculate and draw custom Venn diagrams website (http://bioinformatics.psb.ugent.be/webtools/Venn/).

### Validation of the 55K array in 13 chicken breeds/lines

The genomic DNA from 12 breeds/lines (Chahua, Dagu, Liyang, Luhua, Qingyuan, Silkie, Wenchang, Bai’er, and Xianju, Hubbard, Cobb, and White Leghorn) and two lines with larger populations (Jingxing-Huang and Cobb) were isolated as mentioned above. The genotyping was done on Axiom® arrays using the Affymetrix® GeneTitan® system according to the procedure described by Affymetrix (https://assets.thermofisher.com/TFS-Assets/LSG/manuals/702899_PI.pdf) in the Beijing Compass Biotechnology Co., Ltd. (Beijing, China).

Basic genotype statistics for each marker, including call rate, MAF, Hardy-Weinberg Equilibrium (HWE), allele and genotype counts were calculated using the Quality Assurance Module from the SNP Variation Suite version 7 (SVS; Golden Helix Inc., Bozeman, Montana: www.goldenhelix.com). The following quality control criteria (filtering) were used to remove SNPs with less than 95% call rate for further analysis. The SNPs with less than 0.05 MAF. SNPs were tested for HWE (*P* &lt; 0.001) to identify possible typing error. Samples with more than 10% missing genotypes were removed from the study.

The MDS was performed using the genotype data of the SNPs from the 55K panel on all the breeds samples (*n* = 226) to assess the utility of the panel in detecting population structure. Population structure between 12 breeds was carried out using PLINK software (version 1.90b3) [28] with the MDS method on, and the plot was performed by ggplot2 [35]. The linkage disequilibrium in 2 populations were performed by the GAPIT [36]. The LD decay plot performed by PopLDdecay software are presented as whole genome levels and as chromosome levels with the parameter of smaller break point size of 5 Kb and bigger break point size of 40 Kb [37].

## Additional files

Additional file 1: The summary of each sequencing pools. The raw reads number, clean reads number, sequencing depth, Q30 percentage, and coverage et al. for each sequencing library were provided. (XLSX 14 kb)

Additional file 2: The second group of SNPs which related to the economic traits. The locus and the *p*-value of SNPs which related to 15 economic traits were provided. (XLSX 998 kb)

Additional file 3: The third group of SNPs which related to 861 candidate genes. The information of 861 candidate genes and 118.4 K SNPs selected were provided. (XLSX 6645 kb)

Additional file 4: The third group of SNPs which related to serum IgY. The loci and allele information of 0.8 K SNPs related to serum IgY were provided. (XLSX 32 kb)

Additional file 5: The loci information for the 55K array. The loci, allele information, SNPs frequencies in each breed/line, and overlap information were provided. (XLSX 7761 kb)

Additional file 6: The LD decay in whole genome level in Cobb population. (JPEG 653 kb)

Additional file 7: The LD decay in whole genome level in Jingxing-Huang population. (JPEG 578 kb)

## Abbreviations

CAAS: Chinese Academy of Agricultural Sciences
Chr: Chromosome
Da: inter-population net nucleotide divergence
GLM: general liner model
GWAS: Genome-Wide Association Study
HWE: Hardy-Weinberg Equilibrium
INDEL: insert/deletion.
LD: Linkage Disequilibrium
MAF: Minor Allele Frequency
MDS: Multidimensional Scaling
QTL: Quantitative Traits Loci
RFI: Residual Feed Intake
SNP: Single Nucleotide Polymorphism
UTRs: Untranslated Regions
VEP: Variant Effect Predictor

## References

[1] Resources CNCoAG: animal genetic resources in China: poultry: China agriculture press; 2011.

[2] Ramos AM, Crooijmans RP, Affara NA, Amaral AJ, Archibald AL, Beever JE, Bendixen C, Churcher C, Clark R, Dehais P (2009). Design of a high density SNP genotyping assay in the pig using SNPs identified and characterized by next generation sequencing technology. PLoS One.

[3] Matukumalli LK, Lawley CT, Schnabel RD, Taylor JF, Allan MF, Heaton MP, O'Connell J, Moore SS, Smith TP, Sonstegard TS (2009). Development and characterization of a high density SNP genotyping assay for cattle. PLoS One.

[4] Dash S, Singh A, Bhatia A, Jayakumar S, Sharma A, Singh S, Ganguly I, Dixit S. Evaluation of bovine high-density SNP genotyping Array in indigenous dairy cattle breeds. Anim Biotechnol. 2017:1–7. 10.1080/10495398.2017.1329150 28636460

[5] Anderson R. Development of a high density (600K) Illumina ovine SNP Chip and its use to fine map the yellow fat locus. Plant &amp; Animal Genome. 2014.

[6] Houston RD, Taggart JB, Cézard T, Bekaert M, Lowe NR, Downing A, Talbot R, Bishop SC, Archibald AL, Bron JE (2014). Development and validation of a high density SNP genotyping array for Atlantic salmon ( *Salmo salar* ). BMC Genomics.

[7] Iamartino D, Nicolazzi EL, Van Tassell CP, Reecy JM, Fritz-Waters ER, Koltes JE, Biffani S, Sonstegard TS, Schroeder SG, Ajmone-Marsan P (2017). Design and validation of a 90K SNP genotyping assay for the water buffalo (Bubalus bubalis). PLoS One.

[8] Muir WM, Wong GK, Zhang Y, Wang J, Groenen MAM, Crooijmans RPMA, Megens HJ, Zhang HM, Mckay JC, Mcleod S (2008). Review of the initial validation and characterization of a 3K chicken SNP array. Worlds Poultry Science Journal.

[9] Groenen MA, Megens HJ, Zare Y, Warren WC, Hillier LW, Crooijmans RP, Vereijken A, Okimoto R, Muir WM, Cheng HH (2011). The development and characterization of a 60K SNP chip for chicken. BMC Genomics.

[10] Kranis A, Gheyas AA, Boschiero C, Turner F, Le Y, Smith S, Talbot R, Pirani A, Brew F, Kaiser P (2013). Development of a high density 600K SNP genotyping array for chicken. BMC Genomics.

[11] Derks MFL, Megens HJ, Bosse M, Visscher J, Peeters K, Bink MCAM, Vereijken A, Gross C, Ridder DD, Reinders MJT (2018). A survey of functional genomic variation in domesticated chickens. Genet Sel Evol.

[12] Liu R, Wang H, Jie L, Jie W, Zheng M, Tan X, Xing S, Cui H, Li Q, Zhao G (2017). Uncovering the embryonic development-related proteome and metabolome signatures in breast muscle and intramuscular fat of fast-and slow-growing chickens. BMC Genomics.

[13] Huang HY, Zhao GP, Liu RR, Li SF, Zhao ZH, Li QH, Zheng MQ, Wen J: Expression profiles of novel genes and microRNAs involved in lipid deposition in chicken’s adipocyte. 2017:1–6.

[14] Li P, Fan W, Everaert N, Liu R, Li Q, Zheng M, Cui H, Zhao G, Wen J (2018). Messenger RNA sequencing and pathway analysis provide novel insights into the susceptibility to Salmonella enteritidis infection in chickens. Front Genet.

[15] Fan W, Liu H, Zheng M, Liu R, Li Q, Wen J, Zhao G (2015). Association of BMP15 gene polymorphisms with egg laying in Wuxing-yellow chicken. Chinese Journal of Animal Science.

[16] Liu J, Liu R, Wang J, Zhang Y, Xing S, Zheng M, Cui H, Li Q, Li P, Cui X, et al. Exploring genomic variants related to residual feed intake in local and commercial chickens by whole genomic resequencing. Genes (Basel). 2018;9(2). 10.3390/genes9020057 PMC5852553 29364149

[17] Qi KK, Chen JL, Zhao GP, Zheng MQ, † JW: Effect of dietary ω6/ω3 on growth performance, carcass traits, meat quality and fatty acid profiles of Beijing-you chicken. Journal of Animal Physiology &amp; Animal Nutrition 2010, 94(4):474–485. 10.1111/j.1439-0396.2009.00932.x 19663971

[18] Merat P (1984). The sex-linked dwarf gene in the broiler chicken industry. Worlds Poultry Science Journal.

[19] Aggrey SE, Karnuah AB, Sebastian B, Anthony NB (2010). Genetic properties of feed efficiency parameters in meat-type chickens. Genet Sel Evol.

[20] Wen-bin SJ-t BAO, Cun-bo WANG, Hong-xia ZHANG, Weigend S, Guo-hong CHEN (2008). Investigation on genetic diversity and systematic Evolut ion in Chinese domestic fowls and red jungle fowls by analyzing the mtDNA control region. Acta veterinaria et zootechnica Sinica.

[21] Fu W, Dekkers JC, Lee WR, Abasht B (2015). Linkage disequilibrium in crossbred and pure line chickens. Genet Sel Evol.

[22] Patel RK, Jain M (2012). NGS QC toolkit: a toolkit for quality control of next generation sequencing data. PLoS One.

[23] Li H, Durbin R (2009). Fast and accurate short read alignment with burrows-wheeler transform. Bioinformatics.

[24] Li H, Handsaker B, Wysoker A, Fennell T, Ruan J, Homer N, Marth G, Abecasis G, Durbin R (2009). The sequence alignment/map (SAM) format and SAMtools. Transplant Proc.

[25] Koboldt DC, Chen K, Wylie T, Larson DE, Mclellan MD, Mardis ER, Weinstock GM, Wilson RK, Li D (2009). VarScan: variant detection in massively parallel sequencing of individual and pooled samples. Bioinformatics.

[26] Petr D, Adam A, Goncalo A, Albers CA, Eric B, Depristo MA, Handsaker RE, Gerton L, Marth GT, Sherry ST (2011). The variant call format and VCFtools. Bioinformatics.

[27] Sun Y, Zhao G, Liu R, Zheng M, Hu Y, Wu D, Zhang L, Li P, Wen J (2013). The identification of 14 new genes for meat quality traits in chicken using a genome-wide association study. BMC Genomics.

[28] Purcell S, Neale B, Todd-Brown K, Thomas L, Ferreira MAR, Bender D, Maller J, Sklar P, Bakker PIWD, Daly MJ (2007). PLINK: a tool set for whole-genome association and population-based linkage analyses. Am J Hum Genet.

[29] Liu J, Fu R, Liu R, Zhao G, Zheng M, Cui H, Li Q, Song J, Wang J, Wen J (2016). Protein profiles for muscle development and intramuscular fat accumulation at different post-hatching ages in chickens. PLoS One.

[30] Cui HX, Liu RR, Zhao GP, Zheng MQ, Chen JL, Wen J (2012). Identification of differentially expressed genes and pathways for intramuscular fat deposition in pectoralis major tissues of fast-and slow-growing chickens. BMC Genomics.

[31] Mclaren W, Gil L, Hunt SE, Riat HS, Ritchie GRS, Thormann A, Flicek P, Cunningham F (2016). The Ensembl variant effect predictor. Genome Biol.

[32] Gunderson K, Steemers F, G, Mendoza L, Chee M: A genome-wide scalable SNP genotyping assay using microarray technology. Nat Genet 2005, 37(5):549–554. 10.1038/ng1547 15838508

[33] Syv, Auml AC, nen (2005). Toward genome-wide SNP genotyping. Nat Genet.

[34] Zerbino DR, Achuthan P, Akanni W, Amode MR, Barrell D, Bhai J, Billis K, Cummins C, Gall A, Giron CG (2018). Ensembl 2018. Nucleic Acids Res.

[35] Wickham H (2009). ggplot2: Elegant Graphics for Data Analysis: Springer Publishing Company, Incorporated.

[36] Alexander EL, Feng T, Qishan W, Jason P, Meng L, Peter JB, Michael AG, Edward SB, Zhiwu Z (2012). GAPIT: genome association and prediction integrated tool. Bioinformatics.

[37] Zhang C, Dong SS, Xu JY, He WM, Yang TL. PopLDdecay: a fast and effective tool for linkage disequilibrium decay analysis based on variant call format files. Bioinformatics. . 10.1093/bioinformatics/bty875 30321304

[38] Wang Y, Song F, Zhu J, Zhang S, Yang Y, Chen T, Tang B, Dong L, Nan D, Qian Z (2017). GSA: genome sequence archive. Genomics Proteomics &amp; Bioinformatics.

[39] Members BDC (2018). Database resources of the BIG data center in 2018. Nucleic Acids Res.

