# Supplementary figures and images for: A new chicken 55K SNP genotyping array
Source: BMC Genomics. 2019 May 22;20:410. doi: 10.1186/s12864-019-5736-8 (PMC6532155; doi:10.1186/s12864-019-5736-8)

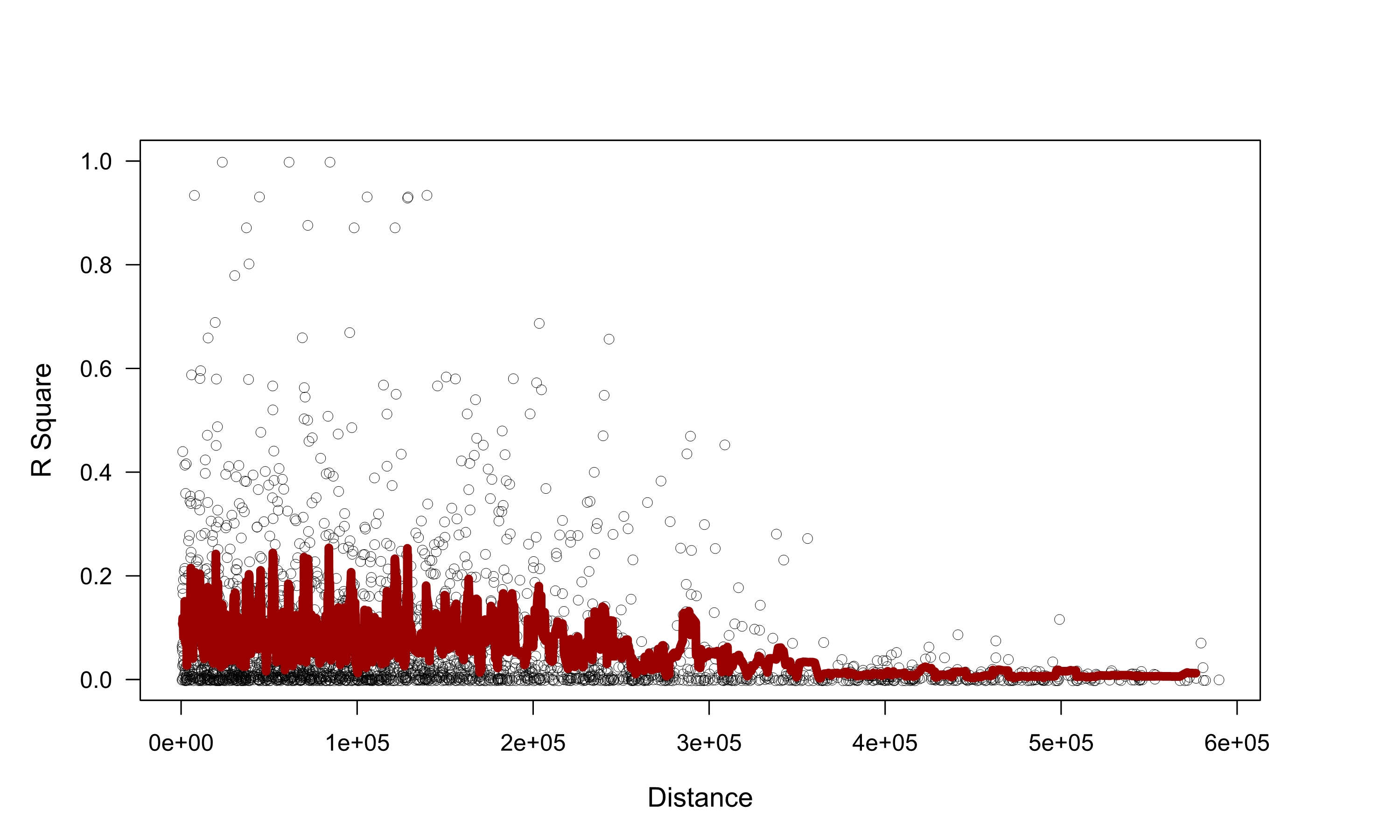

Supplement: Supplementary file 6 — The LD decay in whole genome level in Cobb population. (JPEG 653 kb) [file 12864_2019_5736_MOESM6_ESM.jpeg]

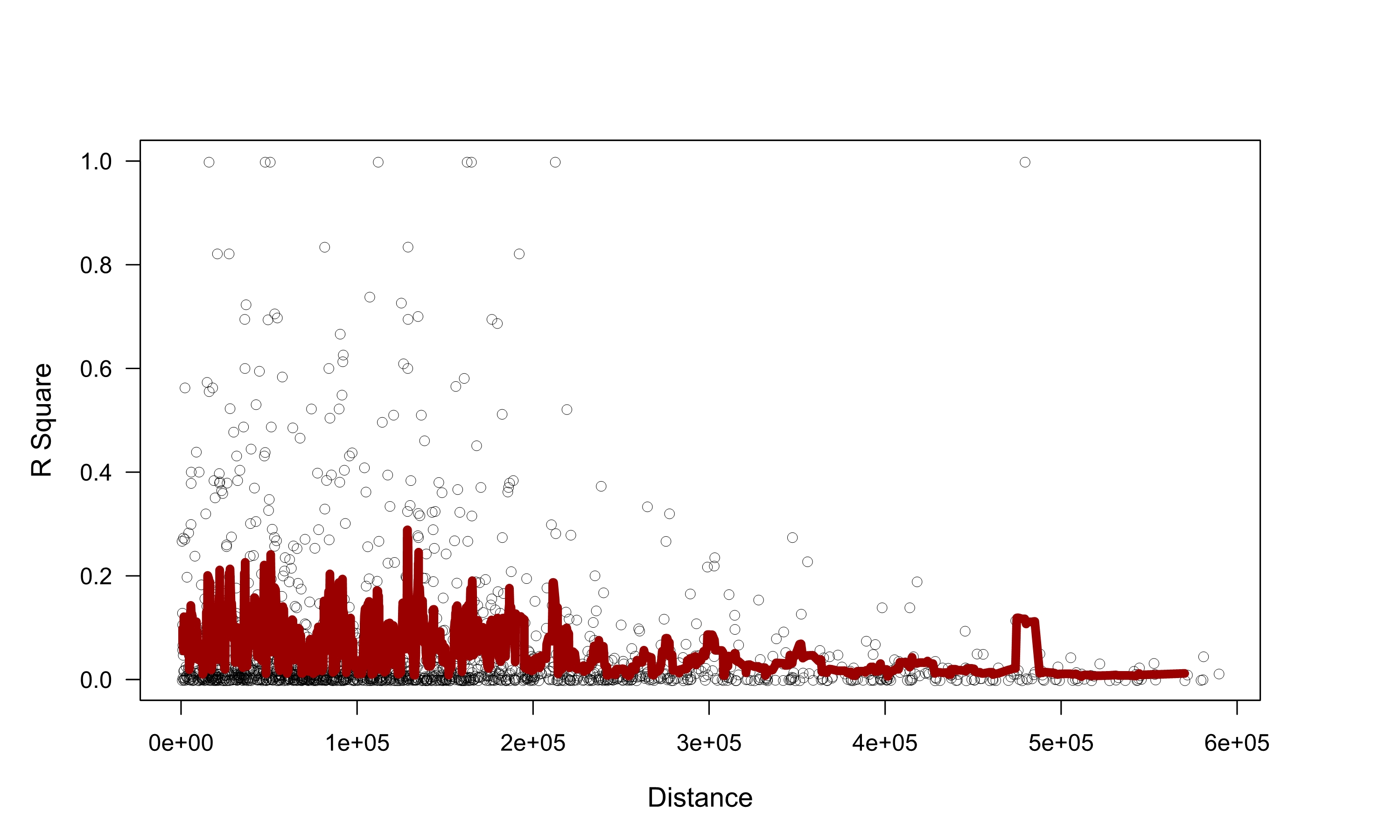

Supplement: Supplementary file 7 — The LD decay in whole genome level in Jingxing-Huang population. (JPEG 578 kb) [file 12864_2019_5736_MOESM7_ESM.jpeg]
